# Effect of traditional Chinese medicine injections on severe pneumonia: A protocol for systematic review and meta-analysis

**DOI:** 10.1097/MD.0000000000022012

**Published:** 2020-09-25

**Authors:** Wei Luo, Ya Liu, Qiang Zhang, Huifang Zhong, Jia Deng

**Affiliations:** a Department of Respiratory and Critical Care Medicine, Chongqing Jiangbei Hospital of Traditional Chinese Medicine; b Department of Dermatology, Chongqing Hospital of Traditional Chinese Medicine; c Department of Oncology, Army Medical Center of PLA; d Department of Personnel Office, Chongqing Jiangbei Hospital of Traditional Chinese Medicine, Chongoing, China.

**Keywords:** meta-analysis, protocol, severe pneumonia, systematic review, traditional Chinese medicine injections

## Abstract

**Background:**

Traditional Chinese medicine injections (TCMJ) used in the treatment of severe pneumonia have been widely implemented in clinical practice, but their overall efficacy and safety remain unclear. This paper aims to evaluate the efficacy and safety of TCMJ in the treatment of severe pneumonia.

**Methods:**

PubMed, EMbase, Cochrane Library, Web of Science, China National Knowledge Infrastructure, WanFang, and the Chongqing VIP Chinese Science and Technology Periodical Database were all searched for randomized controlled trials focusing on the administration of TCMJ for severe pneumonia. Two researchers independently screened titles, abstracts, full texts, and extracted relevant data. The RevMan 5.3 software (The Cochrane Collaboration, Software Update, Oxford, UK) and Stata 14 software (STATA Corporation, College Station, TX) were used for statistical analysis.

**Results:**

This study summarizes the related randomized controlled trials to assess the effect and safety of TCMJ in the treatment of severe pneumonia.

**Conclusion:**

This article provides theoretical support for the clinical application of TCMJ in the treatment of severe pneumonia.

**PROSPERO Registration number:**

CRD42020185072

## 1 Introduction

Patients diagnosed with severe pneumonia, including those caused by the coronavirus disease 2019 have an increased mortality rate.^[1–3]^ Severe pneumonia is a common acute and critical illness mostly encountered in the field of respiratory and critical medicine. It is basically a progressive pulmonary inflammation caused by infection with pathogenic microorganisms, which has the potential of developing acute and severe illness and worsen rapidly.^[4,5]^ Symptoms such as cough, fever, and respiratory distress may occur in the early stage of the disease, and multiple organ dysfunction may arise in severe cases.^[6,7]^ In case the affected patient does not obtain prompt treatment, death is very likely to ensue. Modern medicine often adopts symptomatic treatment measures such as antimicrobial therapy, mechanical ventilation, and homeostatic interventions, but the virulence of various pathogen, together with pathogenic multiple drug resistance seriously affect the clinical efficacy of the treatment regimen, leading to a very uncertain prognosis.^[8,9]^

The role of traditional Chinese medicine injections (TCMJ) in critically ill patients has been gradually discovered since it has been shown to reduce the mortality, shorten the time of mechanical ventilation, and the length of stay in the intensive care unit.^[10–12]^ Over the past couple of years, the efficacy of TCMJ in patients with severe pneumonia has been gradually confirmed by related studies.^[13–16]^ However, the results reported by these clinical trials are still controversial and uncertain. The purpose of this meta-analysis was to evaluate the efficacy and safety of TCMJ in patients with severe pneumonia.

## 2 Methods

### 2.1 Protocol register

This study's protocol has been drafted under the guidance of the preferred reporting items for systematic reviews and meta-analyses protocols.^[17]^ Moreover, it has been registered on PROSPERO (Registration number: CRD42020185072).

### 2.2 Ethics

Ethical approval is not required because there is no patient recruitment or personal information collection, and the data included in our study were extracted from published literature.

### 2.3 Inclusion criteria for research programmes

#### 2.3.1 Type of studies

Randomized controlled trials (RCTs) of TCMJ application for treatment of severe pneumonia were included. Languages are English and Chinese only.

#### 2.3.2 Study object

The study cohort was composed of patients diagnosed with severe pneumonia, regardless of gender, age, race, nationality, and other characteristics.

#### 2.3.3 Intervention type

The control group adopted standard therapy such as antimicrobial therapy, fluid replacement, invasive mechanical ventilation etc. Meanwhile, the experimental group was treated with TCMJ and the type of TCMJ administered (including Xiyanping injection, Xuebijing injection, Reduning injection, Tanreqing injection, Xingnaojing injection, Shenfu injection, Shengmai injection, Shenmai injection) was not limited.

#### 2.3.4 Outcome measurements

Primary outcome:

1) clinical effective rate.

Secondary outcomes:

1) 28-day mortality;
2) length of stay in the ICU;
3) duration of mechanical ventilation;
4) C-reactive protein;
5) procalcitonin;
6) leukocyte count;
7) tumor necrosis factor-α;
8) interleukin-6;
9) D-dimer;
10) adverse reactions.

### 2.4 Exclusion criteria

(1) The literature was published as abstract and conference, and the article's data could not be extracted by contacting the author;
(2) Repeatedly published articles were eliminated and research projects with the most complete information were input;
(3) Articles with either incomplete or erroneous original data were removed;
(4) Articles with obvious randomization mistakes were also excluded.

### 2.5 Search strategy

Retrieval was conducted from PubMed, EMbase, Cochrane Library, Web of Science, China National Knowledge Infrastructure, WanFang and the Chongqing VIP Chinese Science and Technology Periodical Database, for RCTs of TCMJ administration for severe pneumonia cases. The retrieval words used were: traditional Chinese medicine, TCMJ, severe pneumonia etc. PubMed retrieval strategies are displayed in Table 1. We also adapted similar search strategies for other electronic databases.

**Table 1:**
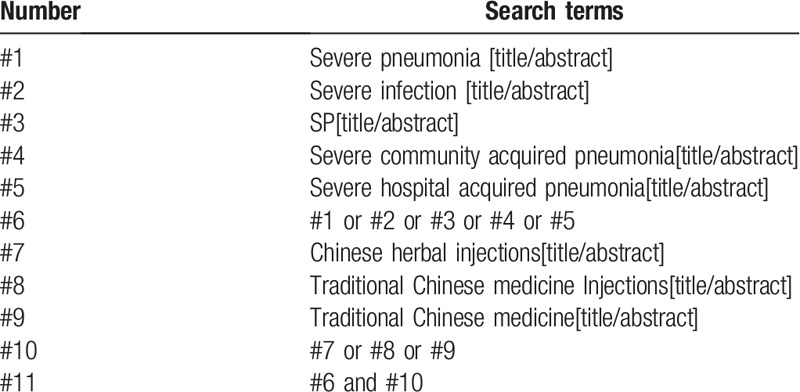
PubMed search strategy.

### 2.6 Data extraction principle

**Figure 1:**
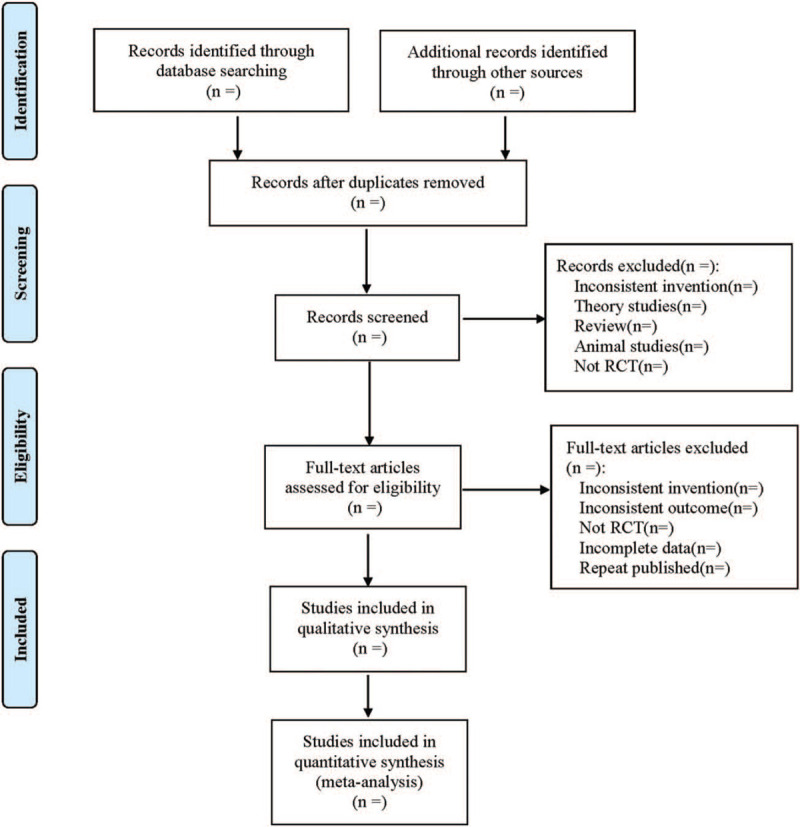
Flow diagram.

Two investigators independently screened the articles. First, we eliminated duplicate pieces of literature. Second, we excluded literature that did not meet the inclusion criteria by reviewing the titles and abstracts. Third, we re-screened the literature's full text that may meet the inclusion criteria to determine whether it was finally included or not, and cross-checked them. Any disagreement was solved via dialogue or discussion with a third investigator when necessary. The 2 investigators independently extracted the data and cross-checked them. Excel 2019 literature information database was established to extract data including the author(s), year of publication, sample size, type of TCMJ, sex, age, intervention measures, course of treatment, outcome, etc. In case of disagreement, the issue was discussed and resolved with a third investigator. The literature selection process is illustrated in Figure 1.

### 2.7 Literature quality evaluation

The 2 researchers independently assessed the risk of bias in the included literature by referring to the Cochrane system reviewer manual. The risk levels of literature quality bias were identified as high, unclear and low. In case of any disagreement, the decision was made after consultation with a third researcher.

### 2.8 Statistic analysis

#### 2.8.1 Data analysis and processing

The RevMan 5.3 software (The Cochrane Collaboration, Software Update, Oxford, UK) and Stata 14 software (STATA Corporation, College Station, TX) were used for statistical analysis. Regarding dichotomous variables, the relative risk was used for statistical analysis. For continuous variables, the Standardized Mean Difference was selected with different tools or units of measurement, and all the above were represented by effect value and 95% Confidence intervals. Heterogeneity test: the Q test was utilized to qualitatively determine inter-study heterogeneity. If *P*≥.1, there was no inter-study heterogeneity. Whereas if *P* < .1, it indicated inter-study heterogeneity. At the same time, the I^2^ value was used to quantitatively evaluate the inter-study heterogeneity. If I^2^≤50%, the heterogeneity was considered to be adequate, and the fixed-effect model was adopted. If I^2^ > 50%, it was considered to have significant heterogeneity, the source of heterogeneity would be explored through either subgroup analysis or sensitivity analysis. If there was no obvious clinical or methodological heterogeneity detected, it would be regarded as having statistical heterogeneity, and the random-effect model would be used for analysis. Descriptive analysis was used if there was significant clinical heterogeneity between the 2 groups and subgroup analysis was not available.

#### 2.8.2 Missing data processing

If data was reported missing or incomplete, we would contact the corresponding author to obtain the missing pieces of information. Otherwise, the concerned study would be be removed.

#### 2.8.3 Subgroup analysis

In order to eliminate the clinical heterogeneity between studies, a subgroup analysis was conducted according to the types of TCMJ administered.

#### 2.8.4 Sensitivity analysis

To test the stability of the meta-analysis results, a one-by-one elimination method was adopted for sensitivity analysis.

#### 2.8.5 Reporting bias

If the included study was ≥10, the funnel plot was used to qualitatively detect publication bias.^[18]^ Subsequently, Egger and Begg tests were used to quantitatively assess the potential publication bias.

## 3 Discussion

Severe pneumonia is an acute and critical disease of the respiratory system, which can further progress and worsened by increased inflammation of the lung tissue, causing multiple organ dysfunction, and can even be life-threatening.^[19]^ In addition to the common respiratory symptoms of pneumonia, severe pneumonia is characterized by insidious development, high mortality, and poor prognosis.^[20]^ In the treatment of severe pneumonia, antimicrobial therapy is the main means to reduce the mortality of patients.^[10]^ Nonetheless, in recent years, with the widespread use of antibiotics and the continuous increase of drug-resistant strains, conventional treatments such as antibiotics are generally unable to achieve an ideal therapeutic effect.^[21]^ Consequently, the most important aspect of the treatment plan is to choose reasonable, effective and safe drugs to control the underlying infection.

TCMJ is the product of the modernization of traditional Chinese medicine dosage form, which has become a unique treatment option for clinical diseases and also plays an irreplaceable role.^[22,23]^ At present, related studies have reported that integrated traditional Chinese and western medicine treatment modalities can safely and effectively reduce the clinical symptoms of patients, as well as lower the mortality and the conversion rate of critically ill patients.^[24,25]^ As a result, traditional Chinese medicine is a potential treatment option for severe pneumonia. In a nutshell, this study systematically evaluates the efficacy and safety of TCMJ in the treatment of severe pneumonia based on the existing pieces of evidence. The presentation of these results provide better treatment alternatives and more convincing evidence for clinical therapy.

Withal, there were some limitations to our study: the included literature only consited of articles written in either Chinese or English, the included literatures are of low quality, allocation concealment and the blinding implementation process was not clear, and the follow-up period was short. The above factors could lead to biased results, so further investigation is still necessary, in order to determine the best way of exploring the safety and efficacy of TCMJ application in the treatment of severe pneumonia. In order to systematically verify the effectiveness and safety of TCMJ in patients with severe pneumonia, it is still imperative to design large sample size, high quality, multi-center RCTs.

## Author contributions

**Data collection:** Wei Luo, Jia Deng.

**Funding support:** Qiang Zhang.

**Literature retrieval:** Ya Liu.

**Software operating:** Huifang Zhong.

**Supervision:** Huifang Zhong.

**Writing – original draft:** Wei Luo, Ya Liu, Jia Deng.

**Writing – review & editing:** Wei Luo, Huifang Zhong.
